# The degradation potential of PET bottles in the marine environment: An ATR-FTIR based approach

**DOI:** 10.1038/srep23501

**Published:** 2016-03-22

**Authors:** C. Ioakeimidis, K. N. Fotopoulou, H. K. Karapanagioti, M. Geraga, C. Zeri, E. Papathanassiou, F. Galgani, G. Papatheodorou

**Affiliations:** 1 Laboratory of Marine Geology and Physical Oceanography, Department of Geology, University of Patras, 26500 Patras, Greece; 2 Institute of Oceanography, Hellenic Centre for Marine Research, 19013 Anavyssos, Greece; 3 Department of Chemistry, University of Patras, 26500 Patras, Greece; 4 Departement Océanographie et Dynamique des Ecosystemes, Institut français de recherche pour l’exploitation de la mer (Ifremer), Bastia, Corsica, France

## Abstract

The dominance and persistence of plastic debris in the marine environment are well documented. No information exists in respect to their lifespan in the marine environment. Nevertheless, the degradation potential of plastic litter items remains a critical issue for marine litter research. In the present study, polyethylene terephthalate bottles (PETs) collected from the submarine environment were characterized using ATR-FTIR in respect to their degradation potential attributed to environmental conditions. A temporal indication was used as indicative to the years of presence of the PETs in the environment as debris. PETs seem to remain robust for approximately fifteen years. Afterwards, a significant decrease of the native functional groups was recorded; some even disappear; or new-not typical for PETs-are created. At a later stage, using the PET time series collected from the Saronikos Gulf (Aegean Sea–E. Mediterranean), it was possible to date bottles that were collected from the bottom of the Ionian Sea (W. Greece). It is the first time that such a study has been conducted with samples that were actually degraded in the marine environment.

Whether we already traversing the “plastic era” that Yarsley and Couzens^1^ aptly described, remains to be seen. It is certain that plastics are ubiquitous in the marine environment, in vast quantities^2^, consisting the major pollutant component of the world’s seas and oceans^3^, present even on the most remote areas of the planet^4^. The boom in the global plastic production, reaching up to 280 million tons in 2012^5^ and the imprudent use of plastics in our everyday life^6^ elevated plastics into a major environmental threat. The generated plastic waste in 192 coastal countries in 2010, has been estimated at approximately 275 million metric tons (MT), from which 4.8–12.7 million MT have entered the oceans^7^.

For the synthetic polymers, the degradation process starts once they are deposited into the oceans, mainly due to the synergistic effect of environmental variables and the inherent material instability^8^. Nonetheless, the degradation rate of the polymers is significantly slow, which makes them extremely persistent^9^. Thus, plastics can last in the marine environment for decades^10^ or even hundreds of years when in surface^11^; likely far longer when in deep sea^12^. Most synthetic polymers (polyethylene, polypropylene) are buoyant in waters while other (high-density polyethylene, polyethylene terephthalate) may sink^13^. The torpid degradation of the large plastic items, will result in the formation of small fragments (<5 mm); microplastics^14^. Both plastics^15^,^16^,^17^ and microplastics^18^,^19^,^20^,^21^ have adverse effects on the marine biota. They may collocate with microorganisms, invertebrates and microbial communities^22^, and even form plastiglomerates^23^.

Microplastics are certainly the most numerically abundant^24^ floating marine litter items, while the larger plastic items (>4.76 mm) seem to prevail in tonnage, roughly estimated weighting approximately 233,500 T (0.33–4.75 mm: 35,000 T)^25^. Especially for the Mediterranean Sea the ranges of floating plastic concentrations are of the same order of magnitude to those recorded in the five subtropical gyres^26^. When it comes to seafloor marine litter, several studies have highlighted them as an important stock deposited on the world’s seafloors^27^,^28^,^29^,^30^. Their behavior and interaction with the deep marine environment is yet unidentified.

Benthic litter tends to become trapped in areas of low circulation and high sediment accumulation. Litter that reaches the seabed may already have been transported considerable distance, only sinking when weighed down by entanglement and fouling. The consequence is the accumulation of litter on specific seabed locations in response to local sources and oceanographic conditions^31^,^32^,^33^,^34^. This is the case of the Saronikos Gulf in Greece, where significant amounts of marine litter tend to accumulate on the deeper zones (200–350 m), reaching densities up to 3,428 items km^−2^ (average: 1211 ± 594 items km^−2^). The vast majority (95%) are plastics, of which the 36.5% counts for plastic bags, 31% for plastic sheets, and 10% for plastic bottles.

Here, we investigate the degradation potential of plastic bottles of different age that were found at the bottom of the Saronikos Gulf (Aegean Sea–E. Mediterranean). The surface properties of plastic PET bottles were investigated in order to gain an insight that may give rise to a hypothesis about the degradation process of plastics in the environment (including the marine).

## Results

**Figure 1:**
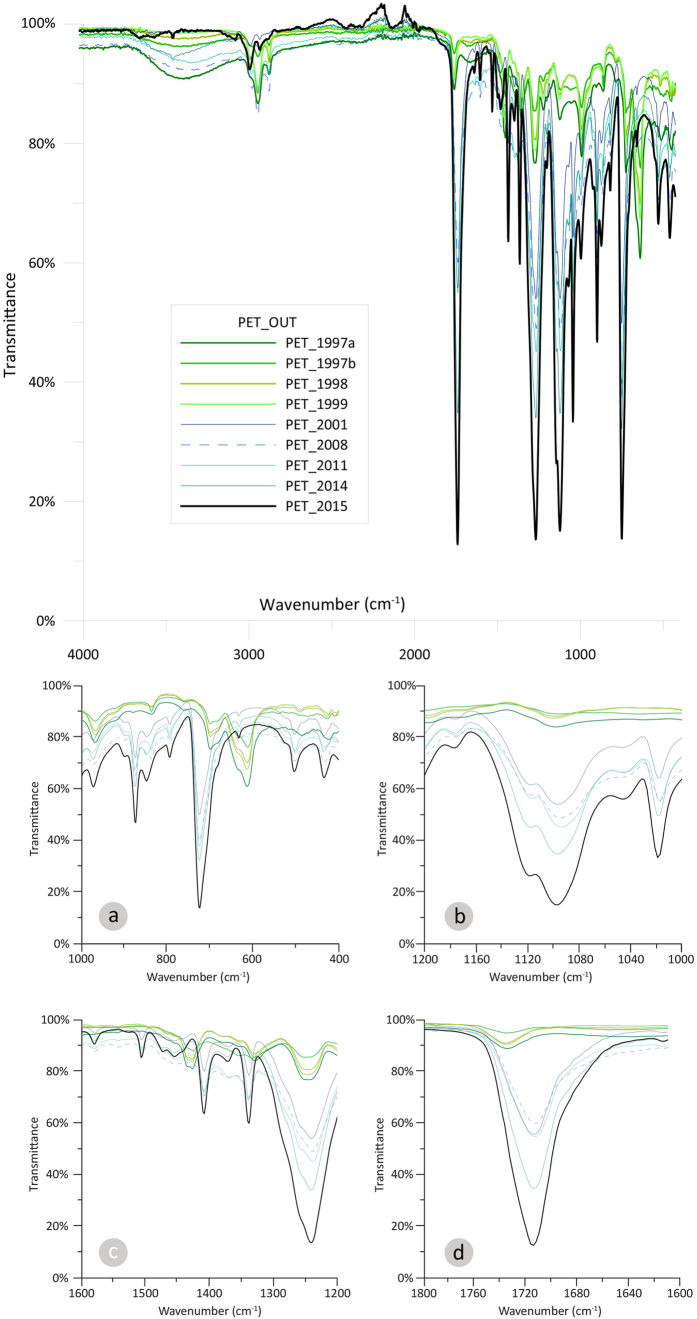
ATR-FTIR comparative spectra of the outer (OUT) surface of the degraded PETs (1997(**a,b**), 1998, 1999, 2001, 2008, 2011, 2014) compared with a virgin sample (2015). For better visualization, enlarged excerpts of the ATR-FTIR comparative spectra are given at wavenumbers (**a**) 400–1000, (**b**) 1000–1200, (**c**) 1200–1600, (**d**) 1600–1800 cm^−1^.

The attenuated total reflectance (ATR)-Fourier transform infrared (FTIR) comparative spectra of the outer surface of the degraded PETs were plotted in a comparative way (Fig. 1). For the PET V, five main peaks are identified at wavenumbers 1715, 1245, 1100, 870, and 730 cm^−1^, corresponding in ketones (C = O), ether aromatic (C-O), ether aliphatic (C-O), aromatic (C-H) and aromatic (C-H) bond. For PET ER, the same peaks are observed. Among the native groups of the polymer (PET), there are various that are decreasing or even disappearing and are attributed to the environmental degradation.

At 1715 cm^−1^ (C = O), there is a decrease; same for 1245 cm^−1^; at 1100 cm^−1^, there is a decrease and in some extent the peak disappears (Fig. 1b); at 870 cm^−1^, the aromatic (C-H) is disappearing, while at 730 cm^−1^, there is a decrease and the peak almost disappears. The outer PET surface also demonstrates new groups, which are not typical for PET. These groups were observed in the samples (1997(a,b), 1998 and 1999) as follows: (a) at 620 cm^−1^ (Fig. 1a) a new alkyne bond (C-H) is created and (b) at 1435 cm^−1^ (Fig. 1c) a new alkane (C-H) bond is created as well.

A similar ATR-FTIR comparative spectra was plotted for the inner PET surface (Fig. S1), with results similar to the ones provided for the outer surface. Thus, the degradation process of PETs seems to happen concurrently for both PET sides i.e. inner and outer surface.

**Figure 2:**
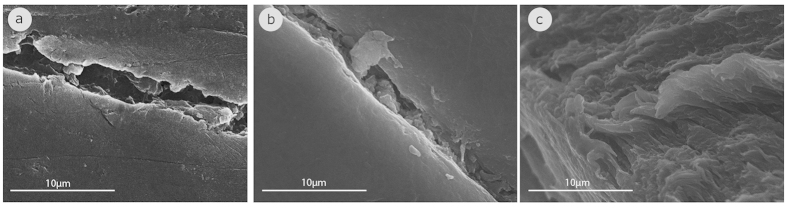
SEM visualizations of the surface topography and roughness of the polymers which are estimated to be present in the environment for more than fifteen years: (**a**) PET_1997a, (**b**) PET_1997b, (**c**) PET_1998.

**Figure 3:**
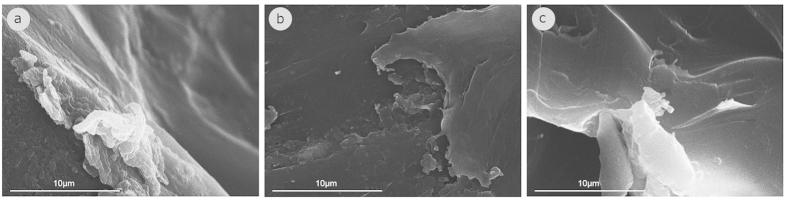
SEM visualizations of the surface topography and roughness of the polymers which are estimated to be present in the environment for less than fifteen years: (**a**) PET_2001, (**b**) PET_2008, (**c**) PET_2011.

Following the investigation of the polymer degradation through the ATR-FTIR comparative spectra, the surface topography and roughness of the polymers were also investigated through SEM visualizations in order to see whether the visualizations give us a similar trend as the ATR-FTIR comparative spectra. The surface topography and roughness of PETs, which are estimated, to be present in the environment for more and less than fifteen years are presented in Figs 2 and 3, respectively. It is obvious that the surface of the newer PETs is smooth whereas the surface of the older PETs is highly cracked and uneven. This is in agreement with the general trend observed by ATR-FTIR that suggests that the surface of the older PETs is altered.

## Discussion

The ATR-FTIR spectroscopy is a common technique that can be used to investigate the environmental degradation of PETs through the determination of any possible changes in the functional groups^35^. While applying this technique to compare materials, special precaution should be taken in respect to the production of false results i.e. decrease in band intensity. In this study, multiple (×5) spectra have been taken for the same spot of each of the three PET bottles (i.e. 1997, 2008, 2015) (Fig. S2). Also, spectra for different spots (×5) of the same PET bottle for each of the three PET bottles (i.e. 1997, 2008, 2015) are shown in Fig. S3. In all these cases, no significant differences between the recorded band intensities have been found compared to spectra differences among PET bottles with different expiration dates. Thus, the presented technique can be reliably used for comparing materials.

Up to our knowledge, this is the first time that plastic items, deposited in the marine environment with a temporal indication, are examined in respect to degradation. The expiration dates on the plastic bottles give an initial estimation regarding the bottles residence time in the environment. Surely, their deposition cannot be safely specified; nonetheless a magnitude regarding the years of presence in the marine environment can be estimated. The age of the bottles was estimated by the marked expiration date. However, we don’t know when exactly the examined bottles ended up in the marine environment; how long were floating in the sea surface or stranded on beach. A conservative estimate regarding the old samples (1997(a,b), 1998, 1999) is that they are exposed in the environment, including the marine environment, for more than fifteen years. When compared to the newer samples (2001, 2008, 2011, 2014), PET surface seems to stay robust for more than fifteen years in the environment. This is corroborated by the additional groups that were created only on the surface of the older samples (1997(a,b), 1998, 1999) at wavenumbers 620 cm^−1^ (C-H) (Fig. 1a) and 1435 cm^−1^ (C-H_2_) (Fig. 1c). It is the first time that such groups are recorded on PETs.

What is really interesting in the finding of the present study is that there is a clear separation of the ATR-FTIR spectra between the two groups i.e. the 90s and the millennium bottles. Thus, giving a distinct sign for the degradation process of PETs. Even for the extreme situation of the 1999 and the 2001 bottles, where there is only a two years interval, still the separation of the ATR-FTIR spectra is very clear.

At the same time, no or scarce information exists regarding the life-time of the synthetic polymers in the environment or under laboratory conditions which could be used as a reference towards the comparison of the present findings. In laboratory experiments studying PET degradation, a life expectancy of PET bottles was predicted under 100% humidity of 27^36^ and 93^37^ years. Whereas Muller *et al.*^38^ in his review paper based on the above-mentioned studies predicted the general life time of PET ranging from 16 to 48 years.

In laboratory studies at low temperatures, hydrolysis appears to be the most important degradation process. The findings of the present study show that plastics seem to follow a different pattern when stranded on the sea floor. Hence, the degradation of plastics is very subjective to the local environmental conditions that are difficult to be simulated in laboratory conditions. It is possible that the initial stage for degradation is the absorption of UV radiation (photolysis). UV light has sufficient energy to break the chemical bonds in the main polymer chain^39^. Then, PETs with altered surface properties sink to the bottom of the sea and hydrolytic degradation starts. Esters are the key functional groups that interact with water^40^. In the old samples, the progress of the degradation becomes slower since the ester functional groups disappear. The creation of new functional groups consisting only of carbon and hydrogen represents a totally new surface that its degradation path is not known.

**Figure 4:**
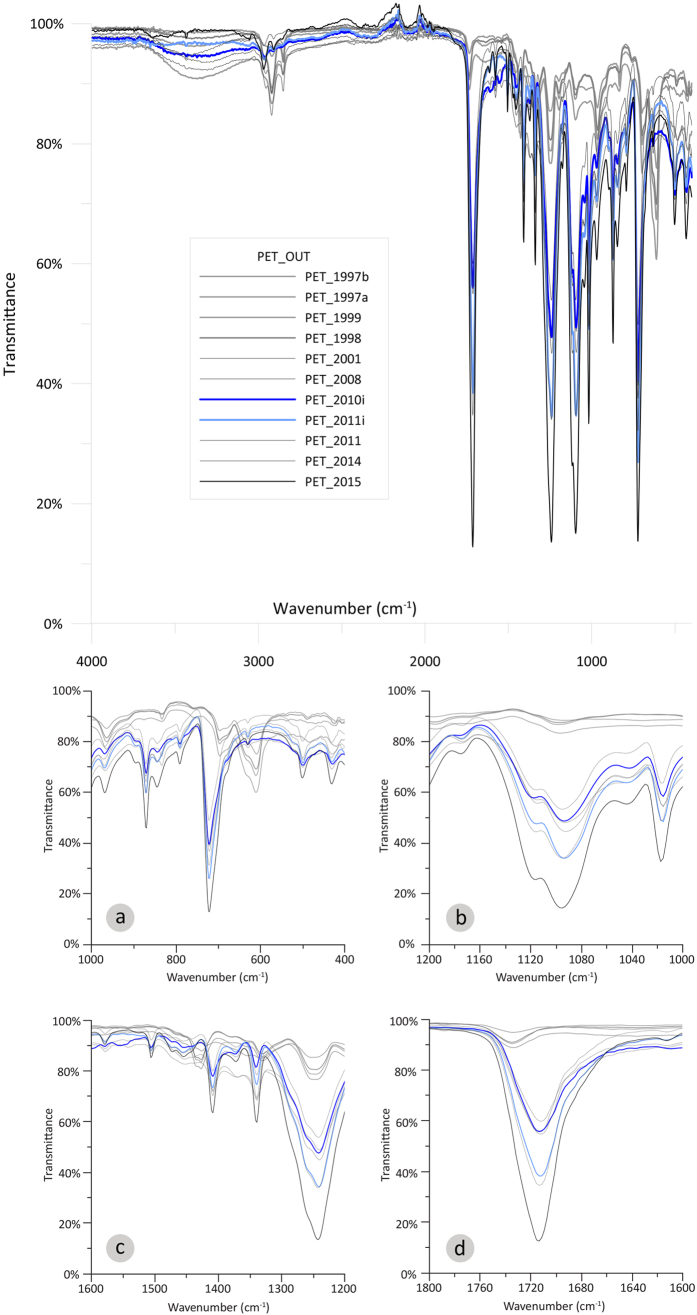
ATR-FTIR comparative spectra of the outer (OUT) surface of the degraded PETs from the Saronikos Gulf (1997(**a,b**), 1998, 1999, 2001, 2008, 2011, 2014) and the old samples from the Ionian Sea (1995i, 1998i) compared with a virgin sample (2015). For better visualization, enlarged excerpts of the ATR-FTIR comparative spectra are given at wavenumbers (**a**) 400–1000, (**b**) 1000–1200, (**c**) 1200–1600, (**d**) 1600–1800 cm^−1^.

In order to confirm whether the severe deterioration of the native functional groups of the PET surface, together with the formation of new ones are denotative of the aged (>fifteen years) PETs and thus could be further used for dating PETs found on the seafloor, the findings of the present study were compared with other PET spectra found in another sea basin. PETs (1995i, 1998i, 2010i, 2011i) from the Ionian Sea (Western Greece) (Fig. S4) were examined in respect to environmental degradation (ATR-FTIR analysis; same rational) and were compared with the ATR-FTIR time-series derived from the Saronikos Gulf (Fig. 4, S5). When the environmental degradation of PETs from the Ionian Sea was compared with that observed in the Saronikos Gulf, the results were very encouraging and confirmed the points discussed above. The two old bottles i.e. 1995i, 1998i from the Ionian Sea gave similar patterns of deterioration for the main five PET peaks at wavenumbers: 1715, 1245, 1100, 870 and 730 cm^−1^ (Fig. 4). Same for the formation of the new functional groups at wavenumbers 620 and 1435 cm^−1^. Especially, the 1998 PET bottle from the Ionian Sea (1998i) gave a similar ATR-FTIR spectrum with that of the 1998 PET from the Saronikos Gulf. Moreover, the ATR-FTIR spectrum of the 1995 PET from the Ionian Sea (1995i) gave even higher deterioration when compared to the oldest PET from the Saronikos Gulf (1997), with the deterioration being significant. The newer samples from the Ionian Sea (2010i, 2011i) followed the same pattern as the corresponding ones from Saronikos Gulf (Fig. S5). Thus by comparing the ATR-FTIR spectra of PET samples, one could date PET litter bottles from different marine environments.

Additionally, by comparing each peak from the Ionian Sea (Fig. 4, S5) with the corresponding peaks (native and new) from the Saronikos Gulf, a first date prediction is attempted for each of the PETs from the Ionian Sea. In Table 1, the date prediction of PETs from the Ionian Sea can be found based on each peak. Based on Table 1, the comparison of all peaks gave a quite accurate prediction for the expiration date of the PET samples from the Ionian Sea. The variation observed is within ± 3 years. Sample 1995i was out of the timeline recorded from Saronikos Gulf, yet the peak comparison suggested that the sample was older than the oldest PET sample from Saronikos Gulf (1997). For sample 1998i, most of the peaks suggested that the sample had an expiration date of 1998 or 1999. For samples 2010i and 2011i, the native peaks predicted their expiration date ± 3 years and the lack of new peaks (620, 1435 cm^−1^) suggested that the samples had an expiration date later than 2001.

The degradation potential of plastics in the marine environment is of crucial importance. Being able to estimate the age of plastics (i.e. PETs) found in the marine environment is critical as it would further help us understand whether plastics have just entered the marine environment or whether they accumulate into the marine environment for decades. Such a finding would further enhance the introduction of management schemes through the understanding of whether the plastic pollution is new or old and tackle the problem accordingly. In addition, such a tool would benefit other scientists involved in studying the effects of marine litter i.e. identification of the age of plastics that have been colonized by organisms or of microplastics that do not have an expiration date written on them. The authors are planning to keep updating the existing database and thus, extending the PET time series along with the introduction of new comparative analytical techniques and methods (such as loss of mass, AFM, TEM, etc.).

## Methods

**Figure 5:**
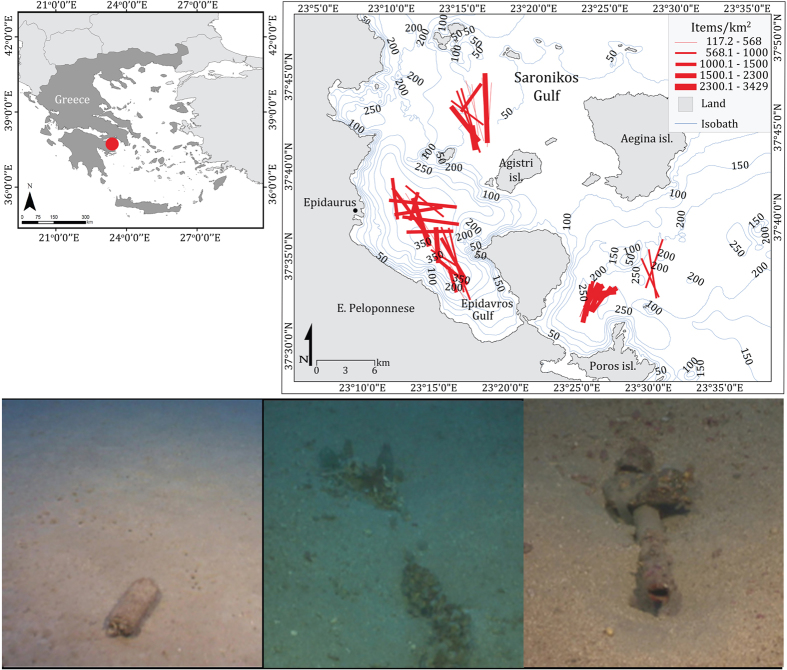
Study map of the Saronikos Gulf (Greece), where the PET bottles were trawled from the submarine environment (ArcMap, ArcGIS 9, V. 9.3; www.esri.com).

On the basis of the assessment of marine litter on the seafloor of the Saronikos Gulf (Greece) from January 2013 to April 2014, plastic marine litter items were collected from its deepest part (150–350 m), from 70 hauls conducted by commercial trawl fisheries (Fig. 5). Plastic bottles made of polyethylene terephthalate (PET) were collected on condition that the expiration date was discernible. From a total of 509 plastic bottles, only in eight^8^ colorless plastic bottles (PETs) the expiration date could be clearly viewable; corresponding in the following years: 1997 (two bottles from the same year; indicated as: a,b), 1998, 1999, 2001, 2008, 2011, 2014 (Fig. S6). The plastic bottles were sorted and coded according to their expiration date, in two categories: the ‘90s bottles (1997(a,b), 1998, 1999) and the millennium bottles (2001, 2008, 2011, 2014). The expiration date-usually two years after production-was used as denotative to the time-period that the samples were present in the environment, including the marine.

All collected samples (PETs) were analyzed in respect to environmental degradation through the estimation of the ATR-FTIR, in order to determine any possible functional groups on the surface of PETs which could be further attributed to environmental degradation. The degraded samples were compared to a similar virgin sample obtained from a supermarket, coded as 2015. Furthermore, the surface topography and roughness as well as the organisms inhabiting on the surface of PETs, were investigated and visualized under SEM.

## Additional Information

**How to cite this article**: Ioakeimidis, C. *et al.* The degradation potential of PET bottles in the marine environment: An ATR-FTIR based approach. *Sci. Rep.* **6**, 23501; doi: 10.1038/srep23501 (2016).

## Supplementary Material

Supplementary Information

## Figures and Tables

**Table 1:** Date predictions for the PET samples from the Ionian Sea based on the comparison with individual peaks recorded for the various PET samples from Saronikos Gulf.

	Ionian Sea PETs			
	1995i	1998i	2010i	2011i
**Native Peaks (cm**^**−1**^)				
1715	1997	1998	2010	2011
1245	<1997	1999	2010	2011
1100	<1997	1999	2008	2011
870	<1997	1999	2010	2008
730	<1997	1999	2008	2011
**New peaks (cm)**				
1435	<1997	1998	>2001	>2001
620	<1997	1998	>2001	>2001
Expiration date	1995	1998	2010	2011
