# Supplementary material for: The degradation potential of PET bottles in the marine environment: An ATR-FTIR based approach
Source: Sci Rep. 2016 Mar 22;6:23501. doi: 10.1038/srep23501 (PMC4802224; doi:10.1038/srep23501)
Supplement: Supplementary Information [file srep23501-s1.pdf]

The degradation potential of PET bottles in the marine environment: An ATR-FTIR based approach.

C. Ioakeimidis[a,b], K.N. Fotopoulou[c], H.K. Karapanagioti[c*], M. Geraga[a], C. Zeri[b], E. Papathanassiou[b], F. Galgani[d], G. Papatheodorou[a]

[a] Laboratory of Marine Geology and Physical Oceanography, Department of Geology, University of Patras, 26500 Patras, Greece.

[b] Institute of Oceanography, Hellenic Centre for Marine Research, 19013 Anavyssos, Greece.

[c] Department of Chemistry, University of Patras, 26500 Patras, Greece.

[d] Departement Océanographie et Dynamique des Ecosystemes, Institut français de recherche pour l'exploitation de la mer (Ifremer), Bastia, Corsica, France.

[*] Corresponding Author. Address: Department of Chemistry, University of Patras, 26500 Patras, Greece; Tel.: +30 2610 996728; e-mail address: karapanagioti@upatras.gr (H.K. Karapanagioti)

SUPPLEMENTARY INFORMATION

1. Materials and methods

On the basis of the assessment of marine litter on the seafloor of the Saronikos Gulf (Aegean Sea – E. Mediterranean) from January 2013 to April 2014, plastic marine litter items were collected from 70 hauls conducted by commercial bottom-trawl fisheries in order to be examined in respect to environmental degradation. Plastic bottles made of polyethylene terephthalate (PET) were collected on condition that the expiration date was discernible. Eight colorless (1997(a-b), 1998, 1999, 2001, 2008, 2011, 2014) plastic bottles (PETs) were collected. The plastic bottles were sorted according to their expiration date. The indicated on the plastic bottles expiration date was used denotative to the time-period that the samples were present in the marine environment.

In the laboratory, samples were rinsed with an electrolyte solution ($NaNO_3$ 0.1 M) to remove all sediment compounds, in order to have a clear polymer surface for investigation. All samples were only handled with laboratory gloves in order to avoid external contamination.

Attenuated Total Reflection (ATR): ATR-FTIR was used in order to determine possible functional groups on the surface of PET, which could be further attributed to environmental degradation. An ALPHA Platinum ATR-FTIR (Bruker Corporation)

with a singly reflection diamond accompanied by the OPUS software was used. For the PET samples, pieces from both inner and outer part of the bottles were selected for analysis.

Scanning Electron Microscopy (SEM): The surface topography, the roughness of the polymers as well as any organisms inhabiting on the surface of the polymers were visualized with a JEOL 6300, Scanning Electron Microscope (SEM). The SEM was equipped with Spectrometers Energy Dispersion X-ray (EDS), Wavelength Dispersion X-ray (WDS) and Cryotrans. The selected for examination surfaces of the PET samples were coated with a layer of gold by sputtering using a JEOL, JFC-1100, ions sputter. Before samples were coated with gold, were rinsed with ethanol solution in order to avoid any kind of contamination. Since the degraded samples were heterogeneous, the visualization was repeated at least three times for each sample, to make sure that a similar image was observed for all degraded samples. Photomicrographs were taken in various fold magnifications (x40, x270, x250, x1000, x1600, x5000, x8000, x8500, x35000) depending on the samples.

2. Supplementary figures

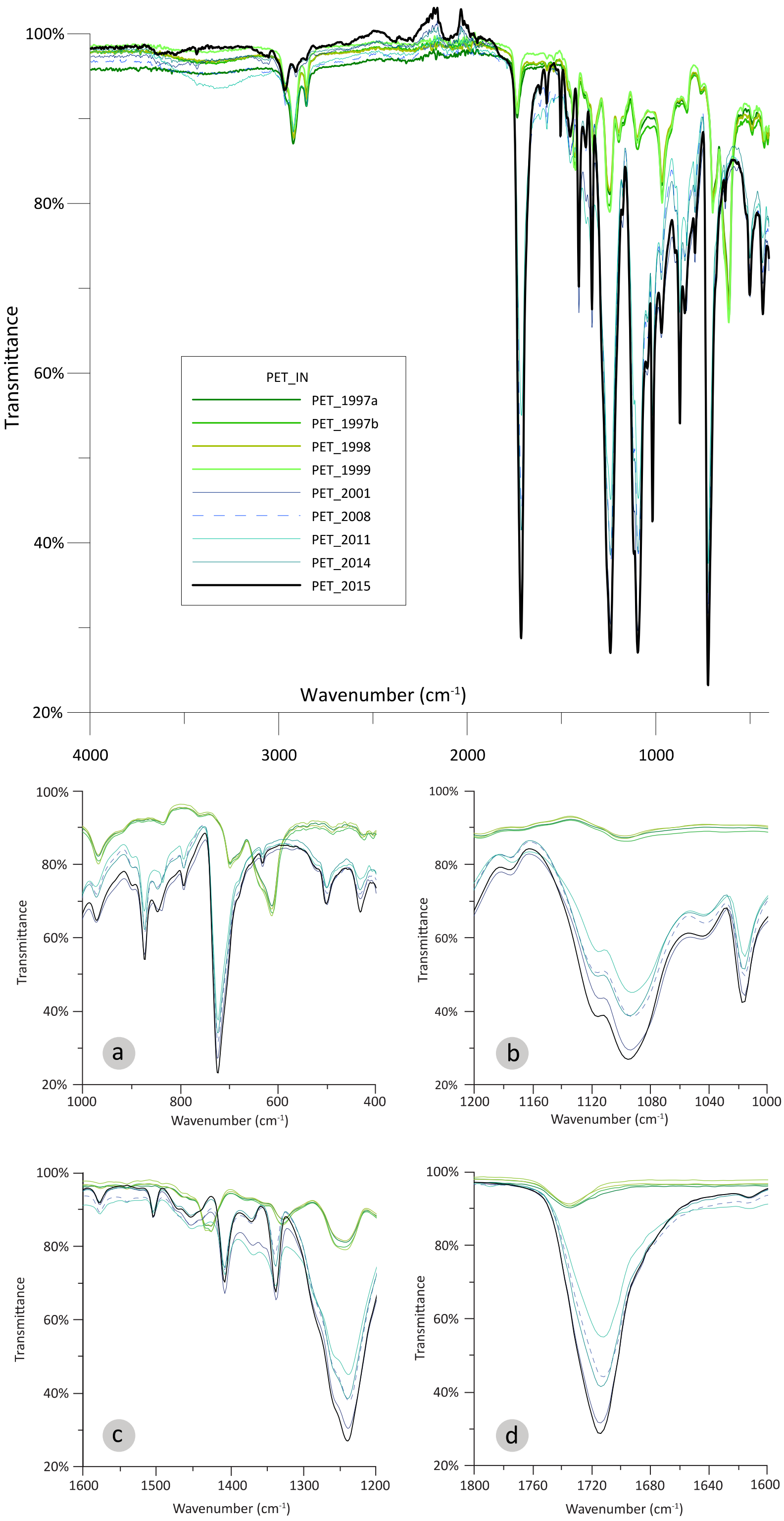

Figure S1: ATR-FTIR comparative spectra of the inner (IN) surface of the degraded PETs (1997(a-b), 1998, 1999, 2001, 2008, 2011, 2014) compared with a virgin (V) sample. For better visualization, enlarged excerpts of the ATR-FTIR comparative spectra are given at wavenumbers (a) 400-1000, (b) 1000-1200, (c) 1200-1600, (d) 1600-1800 cm^{-1}.

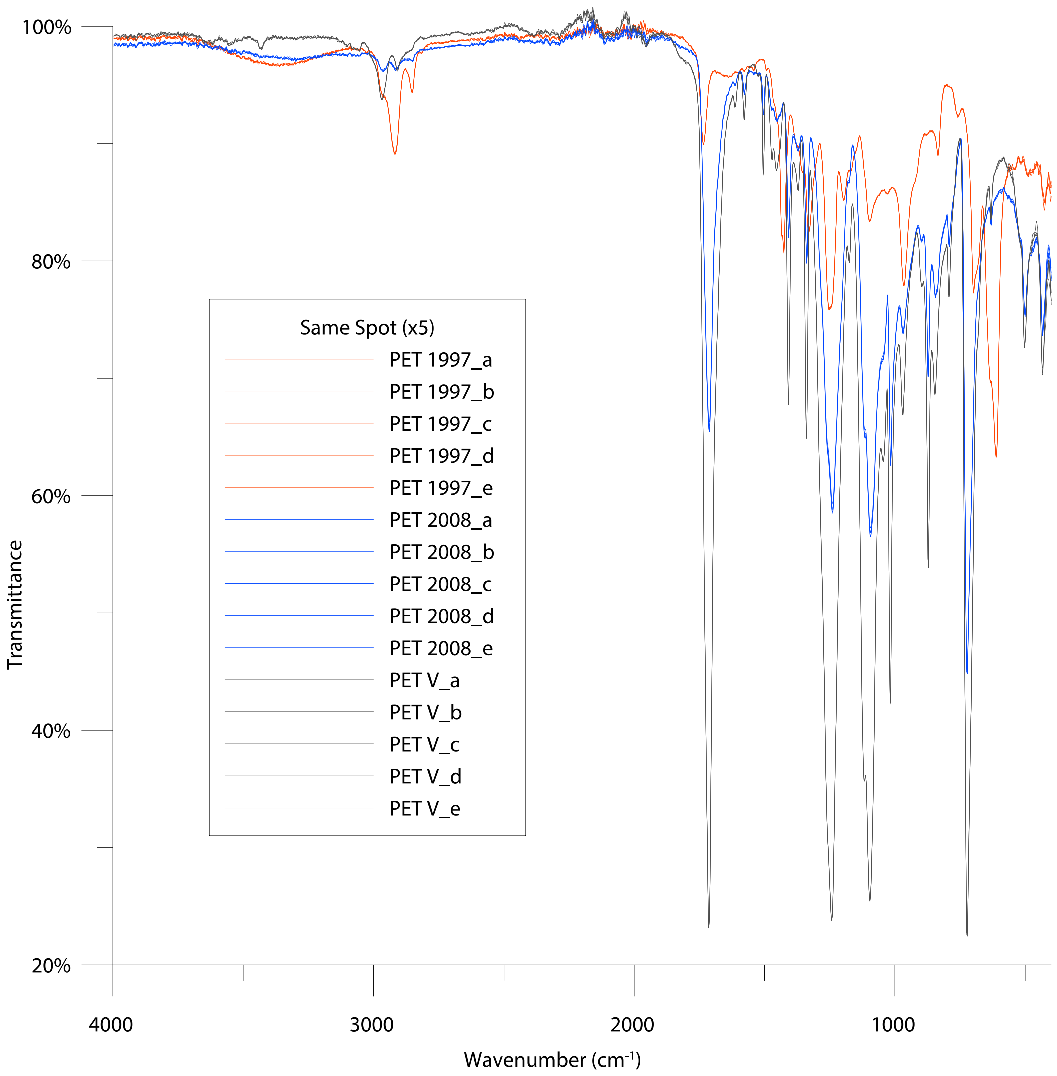

Figure S2: Multiple (x5) FTIR-ATR readings for the same examined spot for the different PET bottles *(1997, 2008 and V)* corresponding in the different groupings (Old – New – V), have been plotted into a single graph in order to visually illustrate whether there is an alteration (i.e. decrease in band intensity) between the readings or not.

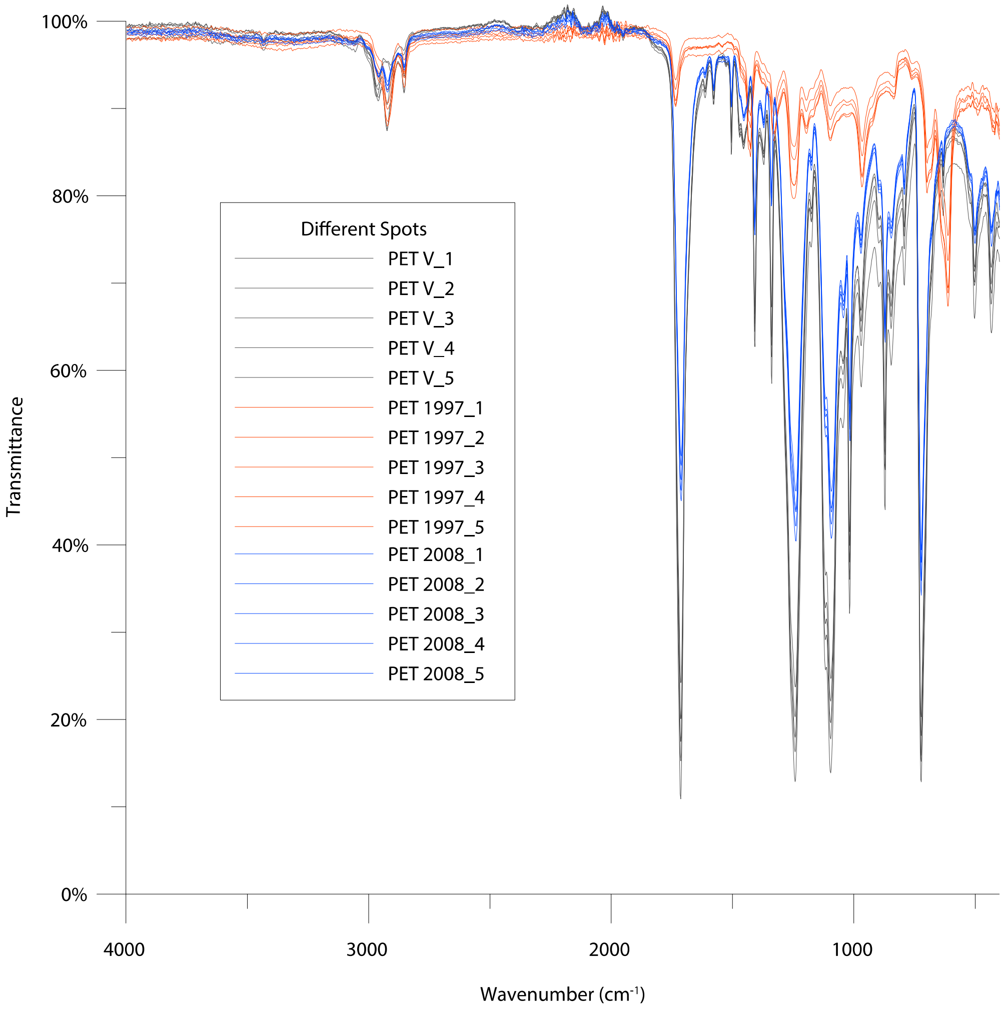

Figure S3: Different FTIR-ATR reading for different spots (#5) for the different PET bottles (*1997, 2008 and V)* corresponding in the different groupings (Old – New – V), have been plotted into a single graph in order to visually illustrate whether there is an alteration (i.e. decrease in band intensity) between the readings or not.

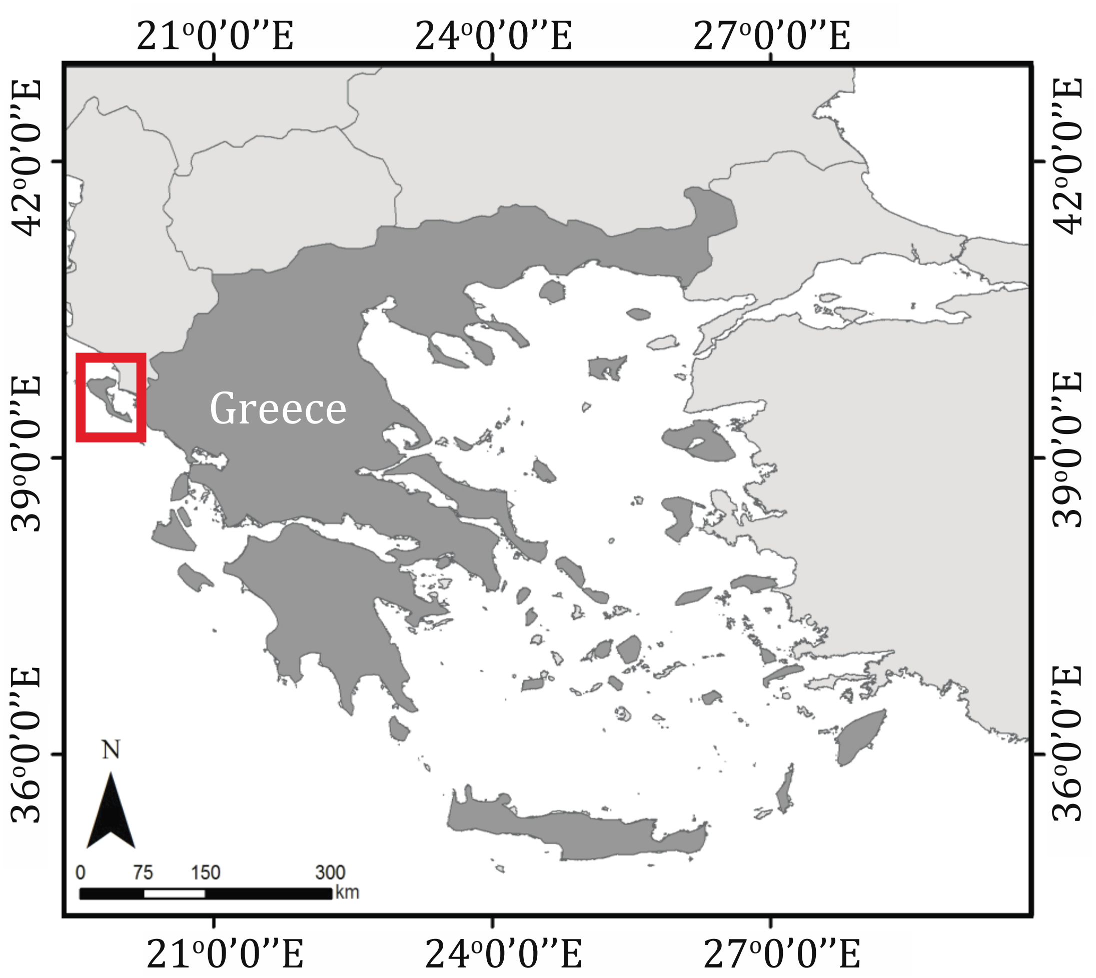

Figure S4: The Ionian Sea (Western Greece) from where the PET samples were used for comparison to those sampled from the Saronikos Gulf (ArcMap, ArcGIS 9, V. 9.3; www.esri.com).

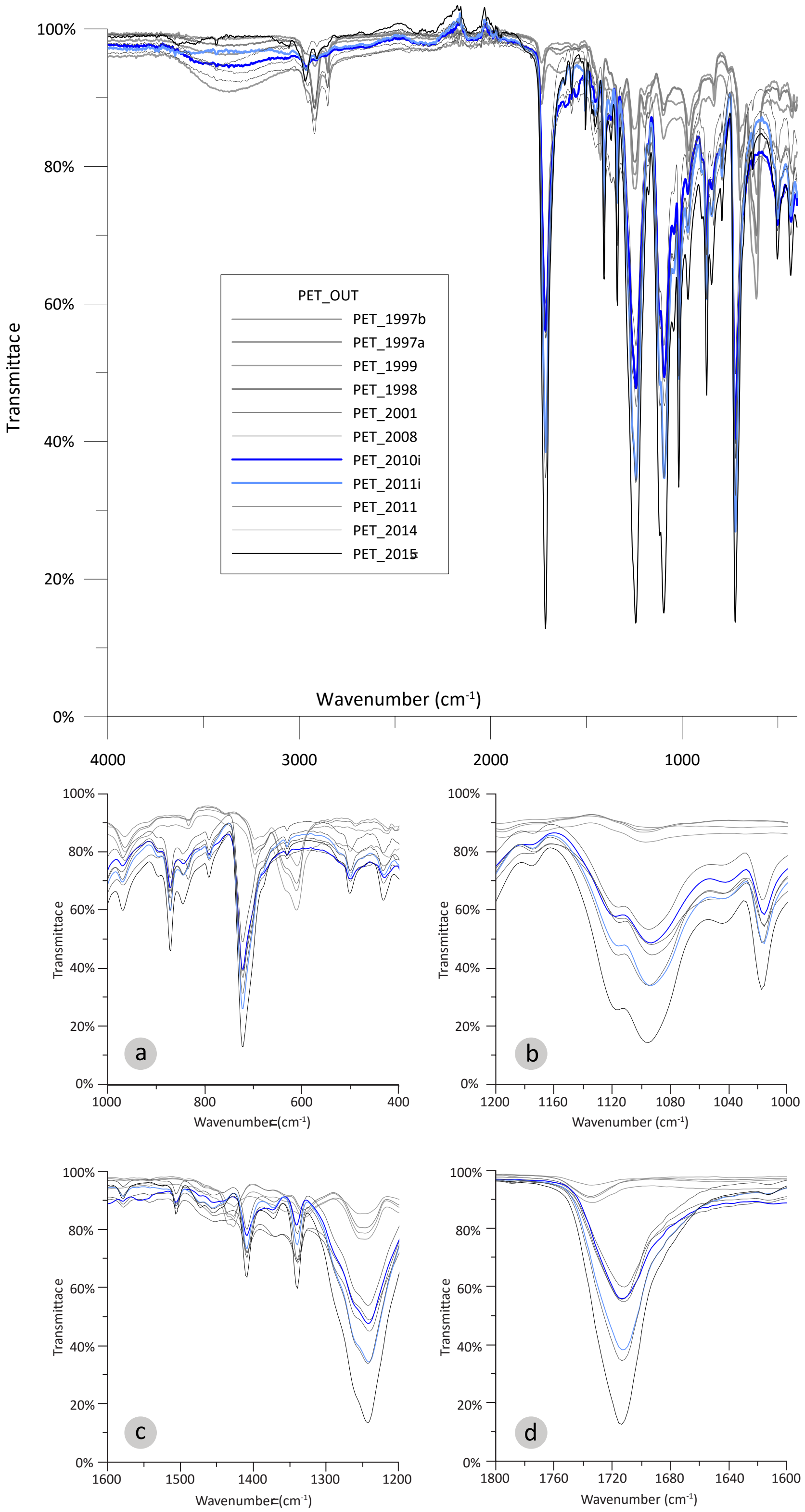

Figure S5: ATR-FTIR comparative spectra of the outer (OUT) surface of the degraded PETs from the Saronikos Gulf (1997(a-b), 1998, 1999, 2001, 2008, 2011, 2014) and the new samples from the Ionian Sea (2010i, 2011i) compared with a virgin (V) sample. For better visualization, enlarged excerpts of the ATR-FTIR comparative spectra are given at wavenumbers (a) 400-1000, (b) 1000-1200, (c) 1200-1600, (d) 1600-1800 cm^{-1}.

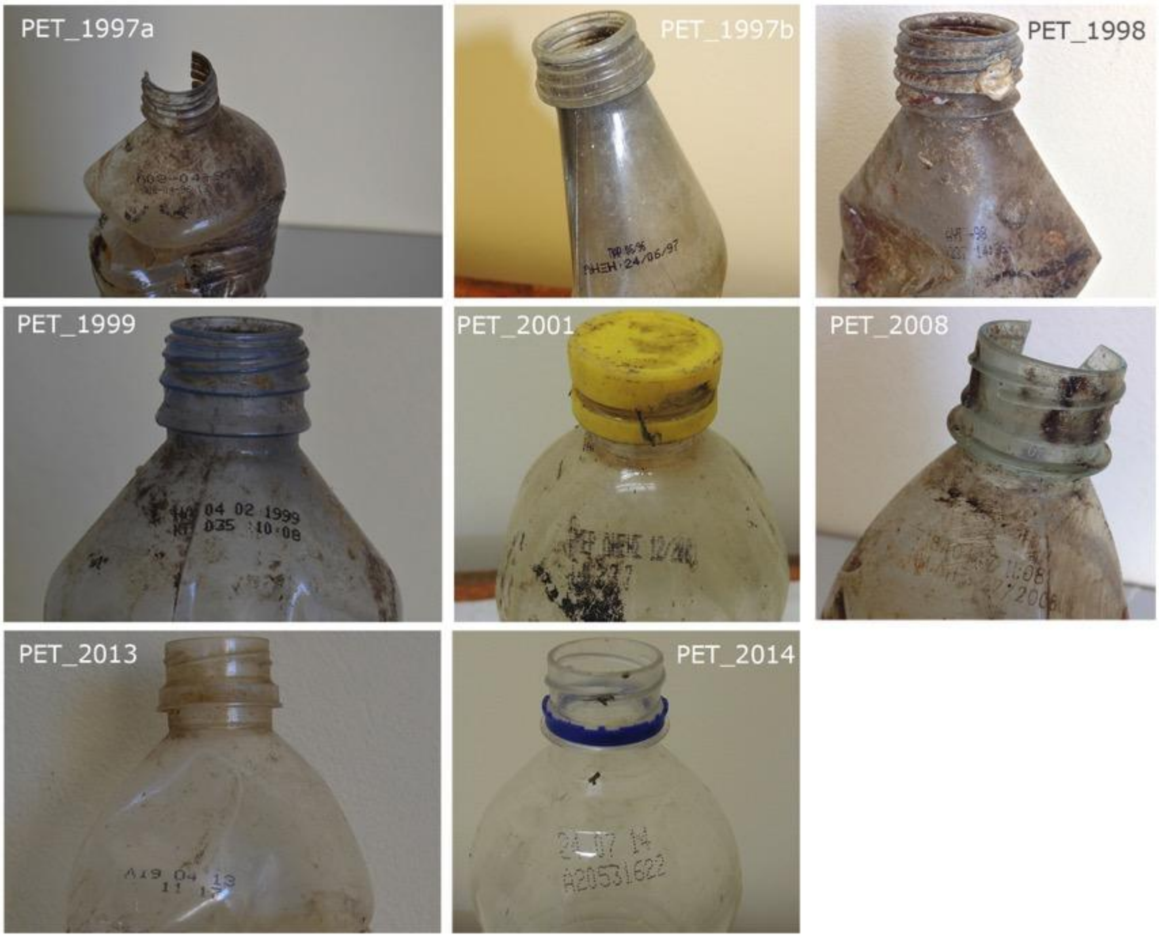

Figure S6: The degraded PETs with a clear indication of their expiration date (1997a, 1997b, 1998, 1999, 2001, 2008, 2013, 2014).
